# Lactic acid production by *Lactobacillus casei* using a sequence of seasonally available fruit wastes as sustainable carbon sources

**DOI:** 10.3389/fbioe.2024.1447278

**Published:** 2024-08-02

**Authors:** Stefania Costa, Daniela Summa, Matteo Radice, Silvia Vertuani, Stefano Manfredini, Elena Tamburini

**Affiliations:** ^1^ Department of Chemical, Pharmaceutical and Agricultural Sciences, University of Ferrara, Ferrara, Italy; ^2^ Department of Environmental and Prevention Sciences, University of Ferrara, Ferrara, Italy; ^3^ Faculty of Earth Sciences, Dep. Ciencia de La Tierra, Universidad Estatal Amazónica, Puyo, Ecuador; ^4^ Department of Life Sciences and Biotechnology, University of Ferrara, Ferrara, Italy

**Keywords:** lactic acid, fruit waste, second cheese whey, fermentation, biorefinery

## Abstract

**Introduction:** Lactic acid (LA) production from fossil resources is unsustainable owing to their depletion and environmental concerns. Thus, this study aimed to optimize the production of LA by *Lactobacillus casei* in a cultured medium containing fruit wastes (FWs) from agro-industries and second cheese whey (SCW) from dairy production, supplemented with maize steep liquor (MSL, 10% v/v) as the nitrogen source.

**Methods:** The FWs were selected based on seasonal availability [early summer (early ripening peach), full summer (melon), late summer (pear), and early autumn (apple)] and SCW as annual waste. Small-scale preliminary tests as well as controlled fermenter experiments were performed to demonstrate the potential of using various food wastes as substrates for LA fermentation, except for apple pomace.

**Results and discussion:** A 5-cycle repeated batch fermentation was conducted to optimize waste utilization and production, resulting in a total of 180.56 g/L of LA with a volumetric productivity of 0.88 g/L∙h. Subsequently, mechanical filtration and enzymatic hydrolysis were attempted. The total amount of LA produced in the 5-cycle repeated batch process was 397.1 g/L over 288 h, achieving a volumetric productivity of 1.32 g/L∙h. These findings suggest a promising biorefinery process for low-cost LA production from agri-food wastes.

## 1 Introduction

Lactic acid (LA) is a well-established and one of the most widely recognized organic acids (Thygesen et al., 2021). Its actual worldwide production of approximately 1.5 million metric tons is anticipated to witness a compounded annual growth rate (CAGR) of 8.2% by 2030 (Ojo and de Smidt, 2023). The global market for LA is rapidly expanding, fueled by food applications as a preservative (Aguirre-Garcia et al., 2024), production of polylactic acid (PLA) as a biodegradable and compostable thermoplastic (Ahmad et al., 2024), and pharmaceutical and personal care applications owing to its antibacterial and detergent properties (Ruiz-Ruiz et al., 2017). Because LA has both carboxylic and hydroxyl functional groups, it can also be considered as a platform and an intermediate for transformation into several different useful and valuable chemicals (Gao et al., 2011).

LA is one of the large-scale compounds for which the biotechnological production has almost completely prevailed through the petrochemical route, with about 90% of the current production achieved by microbial fermentation (Macedo et al., 2020). The fermentative production of LA has been extensively studied for years using a broad range of microorganisms and different types of substrates to optimize yield and productivity (Tian et al., 2021).

The most well-known wild-type LA producers are lactic acid bacteria (LAB), which are non-spore-forming, Gram positive, non-aerobic or aerotolerant, acid tolerant, and strictly fermentative organisms (Fidan et al., 2022). Among the LAB, *Lactobacillus* is the genus of greatest commercial interest as it is homofermentative and produces LA primarily through the Embden–Meyerhoff–Parnas (EMP) pathway by converting one molecule of glucose into two molecules of LA (Singhvi et al., 2018). Recombinant strains of *Escherichia coli, Bacillus coagulans, Corynebacterium glutamicum, Bacillus licheniformis,* and metabolically engineered yeasts have also been evaluated for LA production (Awasthi et al., 2018).

Although industrial-scale biotechnological LA production has been long established, there is room for further process improvements (Abedin et al., 2023). The main obstacle to the use of LAB is their complex nutrient requirement and mesophilia, which lead to increased costs and contamination risks, respectively (Abedi and Hashemi, 2020). Regarding carbon substrates, several agro-industrial low- or no-value wastes, such as molasses, juices wastes, and starchy biomass dairy wastes, have been traditionally fermented into LA (Alexandri et al., 2019; Sakr et al., 2021). More recently, agricultural and forestry residues have also been proposed as carbon sources (Ajala et al., 2020; Yankov, 2022). However, the high costs of the raw materials and fermentation–separation processes as well as selection of highly efficient LA producing microorganisms have severely limited such applications (Ren et al., 2022).

Considerable endeavors have been dedicated to developing fermentation strategies, such as consolidated bioprocesses (CBPs), simultaneous saccharification and fermentation (SSF), and simultaneous saccharification and co-fermentation (SSCF), as promising alternatives (Mazzoli, 2021). To this end, two main concepts have been implemented, i.e., development of synthetic microbial consortia based on co-cultures (Sun et al., 2021) and genetically engineered microorganisms (Levit et al., 2022). In contrast to pure cultures, microbial consortia have been demonstrated to be less vulnerable to environmental disturbances and contamination while exhibiting higher conversion efficiencies (Sun et al., 2019). Nevertheless, the development of reliable methods for co-cultures, growth dynamics, monitoring, and control is still challenging owing to the complex interactions among the microbial population (Mittermeier et al., 2023). Metabolic engineering aims to develop single strains with efficient product formation, yet substantial efforts are needed for major genetic and metabolic redesigning of microorganisms (Hossain et al., 2023).

The second bottleneck for LA production is the overall process cost (Marchesan et al., 2021) from feedstock treatments and sterilization, which are necessary to avoid contamination unless when using thermophilic strains (Garita-Cambronero et al., 2021), to downstream LA production and purification through fermentation. Relative to fermentation, carbon sources are a challenge in terms of cost efficiency as they account for up to 70% of the overall costs (Rawoof et al., 2021). Although refined sugars can theoretically be used as feedstock, with unavoidably lower purification costs as the main advantage, their large-scale industrial use is not economically feasible (Costa et al., 2021). Therefore, wastes and byproducts are preferred because they are inexpensive, abundant, and renewable. Moreover, their use contributes to efficient waste management and reduced environmental pollution in the wider perspective of a circular economy (Walzberg et al., 2021). The downstream processes from the fermentation broth are similarly pivotal to the overall economic sustainability and can account for about 50% of the operational costs (Kumar et al., 2020).

Another limitation to the widespread valorization of byproducts and wastes is related to logistic issues, namely collection and transportation (Caldeira et al., 2020). In fact, one of the intrinsic causes for concern is the environmental impact of transportation over long distances from the waste production to processing site (Read et al., 2020). At the moment, the sidestream residues are suitable raw materials only for small- and medium-scale upgrading or *in situ* valorization in food processing facilities (Vigneshwar et al., 2022). It has been recently estimated that about 30% of global food production is discarded and lost, with 13% lost during the production supply chain and about 17% lost at the consumer level (Ortiz-Sanchez et al., 2023).

Fruit processing industries generate large amounts of wastes, i.e., inedible parts of fruits, damaged or rotten fruit, peel, seeds, pomace, pulp, rinds, and empty fruit bunches, which may account for 20%–80% of the entire processed fruit (Kandemir et al., 2022; Suri et al., 2022). While these are already being employed as animal feed, a major proportion is now destined to be composted or used for biogas production despite more valuable forms of valorization (Ganesh et al., 2022). Some studies have already been published on LA fermentation from fruit wastes (FWs), like using mango peels (Jawad et al., 2013), orange peels (Bustamante et al., 2019), ficus indica wastes (Derabli et al., 2022), tropical fruit byproducts (Ngouénam et al., 2021), and date pulp wastes (Ahmad et al., 2024), as substrates. Whey is the principal residue from the dairy industry (Tsermoula et al., 2021) and consists of cheese whey, which is the byproduct of cheese manufacturing, and second cheese whey (SCW), which is the liquid remaining after further processing of whey cheese that represents more than 90% of the original whey (Pires et al., 2021). Owing to their high organic load and water content, both components can cause severe environmental concerns if not appropriately treated (Poništ et al., 2021). Although several studies have been conducted on cheese whey valorization and a wide range of cheese-whey-derived products are available in the market, SCW has been insufficiently investigated as a substrate for LA fermentation (Sommella et al., 2016). Cheese whey has been earlier proposed as substrate for LA fermentation (Sayed et al., 2020; Zotta et al., 2020; Catone et al., 2021), in addition to biohydrogen (Ordoñez-Araque et al., 2022; Ribeiro et al., 2022) and biofuel production (Osorio-González et al., 2022; Kumar et al., 2023). The use of SCW as a substrate has been attempted for bioethanol (Sansonetti et al., 2009) and biodiesel (Carota et al., 2017) productions; further, it has been used as a substrate for lactobionic acid fermentation using *Pseudomonas taetrolens* (De Giorgi et al., 2018), PHA polymers (Bosco et al., 2021), as an additive in the production of artisanal beers (Pasta et al., 2023), and as a substrate for LAB fermentation for use as probiotics and starters (Secchi et al., 2012). The only example of ovine SCW bioconversion to LA was reported by Secchi et al. (2012), whereas bovine SCW has been used as a substrate to produce LA by Costa et al. (2021) and Dedenaro et al. (2016).

The aim of the present work was to establish LA fermentation by *Lactobacillus casei* using a culture medium based on FWs and SCW as the carbon substrates with maize steep liquor (MSL) as the nitrogen source. MSL is a byproduct of the wet grinding process of maize and is commonly used as a cheap source of nitrogen, amino acids, vitamins, and minerals in fermentation processes (Zhou et al., 2022; Wahjudi et al., 2023). Based on seasonal availability, potential subsequent FW combinations were also investigated. Thus, five main groups were designated in this study, namely, annual (SCW), early summer (early ripening peach), full summer (melon), late summer (pear), and early autumn (apple), to promote full exploitation of the available wastes. Emilia Romagna (Northeast Italy) was identified as the representative region for the present case study given its agri-food production and processing industries.

## 2 Materials and methods

### 2.1 Microorganism and inoculum

The homofermentative *L. casei* (DSMZ 20011) was purchased from the Leibniz Institute DSMZ GmbH (Braunschweig, Germany) and used in this study. The master cell bank was maintained at −20°C in a standard semisynthetic De Man, Rogosa and Sharpe (MRS) medium (1 mL) mixed with glycerol (0.5 mL) as a cryoprotective agent. The standard MRS medium (Fluka Analytical) contained 20 g/L of glucose, 10 g/L of bacteriological peptone, 8 g/L of meat extract, 4 g/L of yeast extract, 5 g/L of CH_3_COONa·3H_2_O, 2 g/L of K_2_HPO_4_, 2 g/L of ammonium citrate tribasic, 0.2 g/L of MgSO_4_·7H_2_O, and 0.05 g/L of MnSO_4_·4H_2_O. The working cell bank was preserved at 4°C in MRS-agar slants for 6 months and used for the seed cultures. All chemicals were purchased from Merck KGaA (Darmstadt, Germany) unless otherwise stated. For the inoculum preparation, *L. casei* was cultured at 30°C and pH = 6.3 for 24 h in sterile MRS medium.

### 2.2 Substrates: SCW and fruit pomace

Fruit pomace (apple, pear, melon, and peach) samples were provided by Conserve Italia (San Lazzaro, Bologna, Italy) based on seasonal availability: early ripening peach (group early summer), melon (group full summer), pear (group late summer), and apple (early autumn); the fruits were harvested and processed in June, July, August–September, and October, respectively. Single raw materials were mixed using a food blender to obtain a thick puree before rough filtration to eliminate the coarser fibrous components as well as skin residues to avoid clogging of the sample pipeline in the fermenter. SCW and MSL were supplied by Granarolo Spa (Bologna, Italy) and Cargill Spa (Calstelmassa, Rovigo, Italy), respectively. MSL supplement was used as an alternative to yeast extract or peptone as the nitrogen source. All materials were stored in plastic tanks at −18°C for laboratory analyses and use as fermentation substrates.

### 2.3 Apple pomace filtration and hydrolysis

Apple pomace was subjected to two alternative pretreatments: filtration and enzymatic hydrolysis. Filtration was performed with a mechanical filter with a 5-mm-sieve via gravity at 4°C; approximately 300 mL of apple filtrate was recovered and analyzed for the sugar content. Enzymatic hydrolysis was performed using a commercial enzyme mixture (Celluclast™, Novozymes) containing cellulase from *Trichoderma reesei* and β-glucosidase from *Aspergillus niger*. Assays were conducted in 250 mL Erlenmeyer flasks at 50°C under agitation (180 rpm) for 12 h, with the addition of 0.05 M citrate buffer (pH = 4.8). Then, approximately 2 mL of the enzyme suspension at a declared activity of 700 endoglucanase units (EGU)/g was added to 150 mL of the apple pomace sample (Zhang et al., 2017); the apple pomace hydrolysate was then analyzed for its sugar content.

### 2.4 Single substrate fermentation

Single substrate fermentations were performed for preliminary assessments of the fermentative capacities of *L. casei* on the selected carbon sources to define the baseline for further process development and to study the feasibility of sequentially using the various feedstocks. The fermentations were carried out in 100 mL Erlenmeyer flasks with 100 mL working volume. Approximately 100 mL of the various substrates, i.e., SCW, pear pomace, peach pomace, apple pomace, and melon wastes, were added to 10 mL of MSL and inoculated with 10% (v/v) of an exponentially growing inoculum of *L. casei* at 30°C and pH = 6.3 for 96 h at a stirring rate of 100 rpm on a rotary shaker for growth as single carbon sources. The samples were withdrawn at 0, 24, 48, 72, and 96 h, before being filtered and stored at 4°C until the analyses. All experiments were carried out in triplicate, and the standard deviation is reported as the measure of errors.

### 2.5 Sequential repeated batch fermentation

**FIGURE 1:**
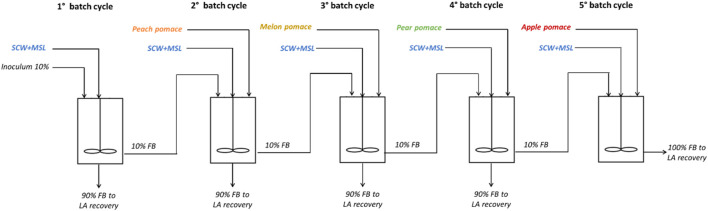
**-** Experimental scheme of the sequential repeated batch fermentation process based on seasonally available FWs, including SCW as an annual available waste and MSL as the nitrogen source (FB, fermentation broth).

Repeated batch fermentation processes were carried out by considering five batch cycles (Figure 1), each using a different FW depending on seasonal availability, with SCW as the substrate available throughout the year and MSL as the nitrogen source. A thermoregulated autoclavable Minifors^TM^ bioreactor (Infors, Basel, Switzerland) (1 L working volume, 1.5 L overall capacity) equipped with probes for the pH, temperature, and dissolved oxygen (Mettler Toledo, Columbus, United States) was used. The pH values of the cultures were automatically maintained at 6.3 by the addition of 1 N NaOH solution.

Before inoculation, the fermenter was filled with a mixture of SCW and 10% MSL along with the various FWs at appropriate percentages depending on the cycles. The culture was stirred at 50 rpm with a mechanical stirrer. An airflow of 0.5 L/min was fluxed on the head space of the fermenter to preserve a slight overpressure. Each cycle was followed for 48 h and samples were retrieved at 0, 6, 12, 24, 36, and 48 h. About 90% of the working volume was discharged at the end of each cycle, while 10% of the fermentation broth from each cycle was retained in the fermenter as inoculum for the following cycle. The following ratio of substrates was added to the SCW:

- 1^st^ batch: SCW-MSL 100% + FW 0%
- 2^nd^ batch: SCW-MSL 30% + FW (peach pomace) 70%
- 3^rd^ batch: SCW-MSL 30% + FW (melon pomace) 70%
- 4^th^ batch: SCW-MSL 30% + FW (pear pomace) 70%
- 5^th^ batch: SCW-MSL 30% + FW (apple pomace) 70%

### 2.6 Analytical assays

Sugars (glucose, fructose, sucrose, and lactose) were analyzed by high-performance liquid chromatography (HPLC; Jasco, Easton, MD, United States) equipped with refractive index and UV detectors (Jasco, Oklahoma City, OK, United States) as well as Rezex ROA-Organic Acid H+ (8%), 300 × 7.8 mm (Phenomenex, Torrance, CA, United States). Isocratic elution was conducted at 30°C with 0.6 mL/min of 0.01 M H_2_SO_4_. Before the HPLC analyses, the samples were centrifuged (6720 RCF; 10 min), maintained at 80°C for 10 min to eliminate possible interferences due to the microbial enzymes, and filtered using cellulose acetate filters (porosity of 0.2 µm). The overall yield of LA was calculated as the gram-produced LA per gram of available total sugars and gram per consumed total sugars. The maximum production rate was calculated as the mass of the maximum LA production per volume of the fermentation broth in time units (hours).

## 3 Results

### 3.1 Sugar contents of the substrates

**FIGURE 2:**
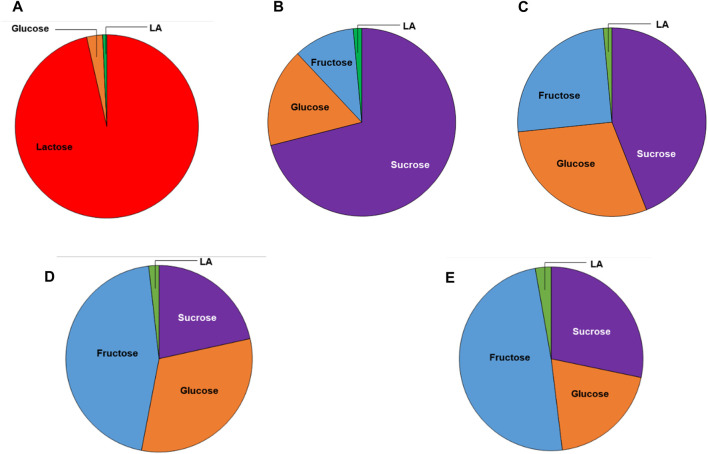
Pie charts of the sugar compositions (on a wet basis) of the substrates used in this study: **(A)** SCW; **(B)** peach pomace; **(C)** melon pomace; **(D)** pear pomace; **(E)** apple pomace. The green slices correspond to the initial LA concentrations due to spontaneous fermentation.

**FIGURE 3:**
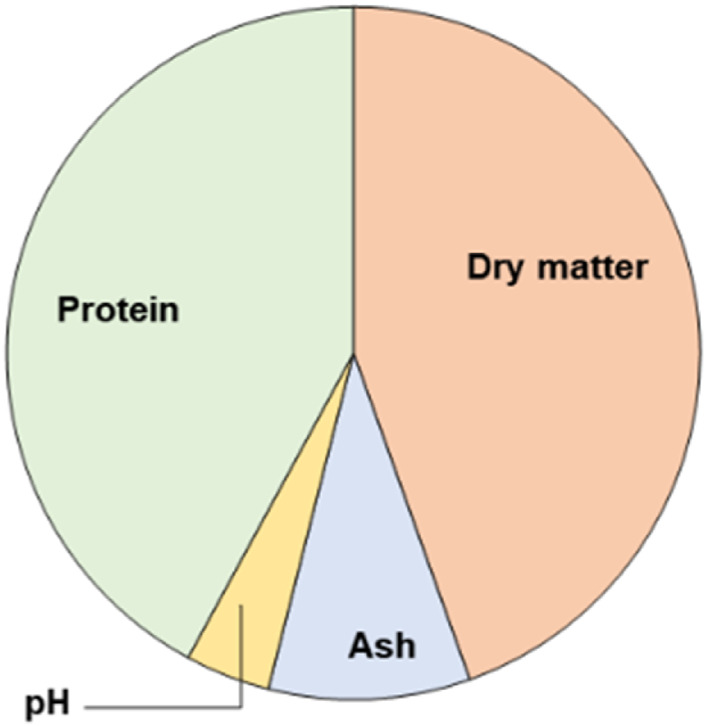
Pie charts of MSL compositions (on a wet basis).

The sugar contents of the different substrates used in this study are reported in Table 1 and Figure 2. The total sugar content of the FWs were 29.3 ± 1.21, 28.7 ± 0.87, and 58.2 ± 4.4 g/L for the melon, pear, and apple pomace, respectively, and 40.4 g/L for the peach pomace. The SCW sugar content was 46 g/L, and this amount was almost entirely due to lactose. The differences in the sugar contents of the FWs are attributable to the different compositions of pomace; peach pomace is principally composed of rotten fruit pulp without the peach pits and skins, which are usually removed by chemical treatment of the whole fruit before processing in the industry, except for fresh peaches destined for fresh consumption. Conversely, melon, pear, and apple pomace samples included the skins, kernels, stalks (for apple and pear), and thick peel (for melon), as well as all fibrous parts that decreased the fermentable sugar contents. The chemical composition of MSL was provided by the manufacturer (Table 2; Figure 3), and it was analyzed here for determination of the sugar and LA contents.

**TABLE 1:** Sugar compositions of the raw materials on a wet basis.

Raw material	Sucrose (g/L)	Lactose (g/L)	Glucose (g/L)	Fructose (g/L)	LA (g/L)
SCW	—	44.67 ± 1.78	1.30 ± 0.09	—	0.34 ± 0.02
Peach pomace	29.10 ± 2.62	—	6.96 ± 0.49	4.31 ± 0.17	0.60 ± 0.03
Melon pomace	13.10 ± 0.52	—	8.73 ± 0.17	7.49 ± 0.52	0.44 ± 0.02
Pear pomace	6.30 ± 0.21	—	9.22 ± 0.29	13.21 ± 0.37	0.52 ± 0.02
Apple pomace	16.92 ± 1.23	—	11.85 ± 0.69	29.41 ± 2.48	1.67 ± 0.13
MSL	—	—	3.5 ± 0.24	—	2.50 ± 0.12

**TABLE 2:** Chemical composition of MSL (as supplied by the manufacturer).

MSL	%
Dry matter	45.0 ± 7.0
Ash	9.5 ± 2.5
pH	4.0 ± 1.0
Protein (as Nx6.25 on d.b.^a^)	42.5 ± 6.5

^a^ Dry basis.

A small quantity of LA was already present in all FWs and SCW, probably due to the initial LA fermentation over the recovery, storage, and transportation durations probably due to the high summer temperatures. The occurrence of LA in MSL is well-established (Nisa et al., 2006; Kaur et al., 2020). The filtered apple pomace sample was characterized by a lower sugar content than apple pomace as a relevant amount of the soluble sugars remained in the filtration cake (Table 3). On the contrary, significant increases of glucose and fructose were evidenced in the hydrolysate, confirming the high amounts of polysaccharides, i.e., glucan, starch, and cellulose, in apple pomace (Gullón et al., 2008).

**TABLE 3:** Sugar compositions of pretreated apple pomace on a wet basis.

Raw material	Sucrose (g/L)	Lactose (g/L)	Glucose (g/L)	Fructose (g/L)	LA (g/L)
Apple pomace filtrate	4.43 ± 0.22	—	4.87 ± 0.37	8.42 ± 0.52	—
Apple pomace hydrolysate	—	—	88.94 ± 2.98	147.05 ± 5.98	0.32 ± 0.00

### 3.2 Fermentation and LA production by *L. casei* on non-hydrolyzed single substrates

**FIGURE 4:**
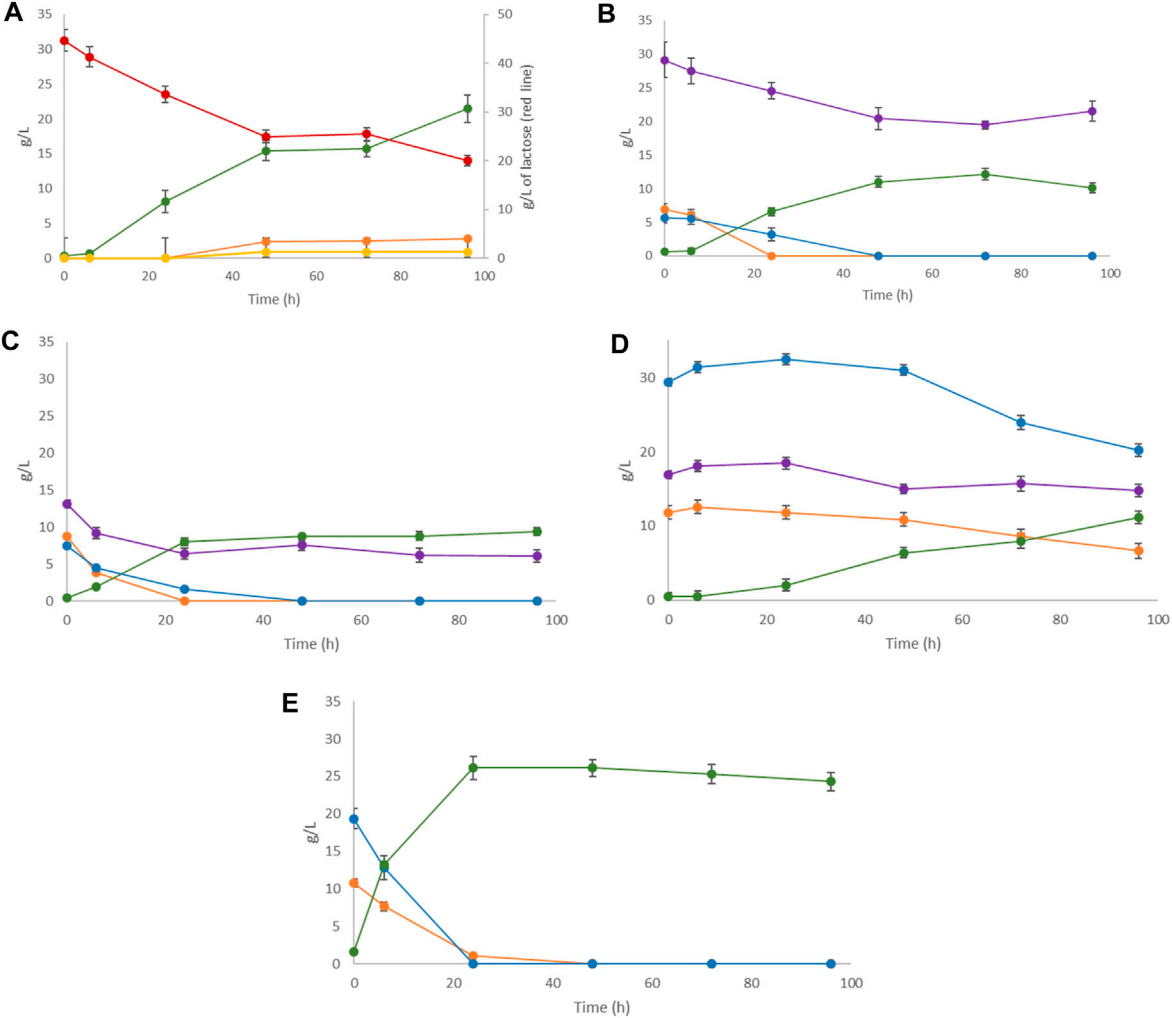
Single substrate LA fermentation by *Lactobacillus casei* with **(A)** SCW, **(B)** peach pomace, **(C)** melon pomace, **(D)** apple pomace, and **(E)** pear pomace as the carbon sources. Concentrations of LA (green line) along with lactose (where present, red line), sucrose (purple line), galactose (where present, yellow line), glucose (orange line), and fructose (blue line) were monitored for 96 h.

The capacity of *L. casei* to grow and produce LA on FWs was tested preliminarily on the single substrates. LA production from SCW by *L. casei* has already been established in a previous work (Costa et al., 2021), along with microbial growth on MSL. The FW samples were only ground and homogenized because they contain promptly fermentable sugars as mono- and di-saccharides. Figure 4 shows the sugar depletion and LA production after 96 h of fermentation of each substrate.

As seen, the LA yield was generally low due to the fact that the pH cannot be regulated in small-scale tests and spontaneously tends to reach acidic values, jeopardizing the biomass growth and provoking the cessation of fermentation. In fact, the main aim of these tests was to exclude the presence of toxic substances that could inhibit microbial growth, rather than optimizing the LA yield. The small-scale preliminary trials showed that all FWs had promising potential as substrates for LA fermentation except for apple pomace, as evidenced by the slow and low sugar utilization by *L. casei.* Of the overall sugar content (58.2 g/L), only 28% was consumed with low LA production (less than 20% yield from the initial sugar content) (Table 4). Apple pomace is mainly composed of skin, flesh (95%), and seeds (2%–4%) (Kammerer et al., 2014) and is characterized by its high content of polysaccharides, such as gluco-, xylo-, and arabino-oligosaccharides (Gullón et al., 2008), and polyphenols, such as procyanidin B2 and chlorogenic acid, that should act as growth stimulants for LAB (Duda-Chodak and Tarko, 2023). Attempts to improve the process efficiency of apple pomace have focused on the introduction of filtration or hydrolysis pretreatments prior to fermentation.

**TABLE 4:** Total sugar (TS, g/L), residual sugar (RS, g/L), and LA yields on TS (Y_LA/TS_) and consumed sugar (CS) (Y_LA/CS_) for single carbon source fermentations by *L. casei*.

Substrate	TS (g/L)	RS (g/L)	Y_LA/TS__(%)_	Y_LA/CS__(%)_
SCW	45.97	20.92	48	99
Peach pomace	40.37	21.74	25	54
Melon pomace	29.32	6.07	32	40
Apple pomace	58.18	41.74	19	68
Pear pomace	28.73	0	85	85

*L. casei* fermentation on lactose clearly showed a strong dependence on product inhibition probably due to the concentration of LA, which resulted in a decrease in pH. When the pH is lower than the pKa value of LA (3.86), LA can diffuse into the cytoplasm through the plasma membrane and dissociate into lactate and protons (Maris et al., 2004). This leads to acidification of the cytoplasm and disruption of the proton motive force, resulting in inhibition of nutrient transport (Singhvi et al., 2018). Analogous effects were observed during growth for the peach and melon pomace samples after approximately 50 h of fermentation. In these cases, complete depletion of glucose and fructose was achieved, whereas sucrose remained unfermented. *L. casei* shows very short lag phases in all FW substrates (<6 h), as confirmed by Rezvani et al. (2017).

### 3.3 Fermentation and LA production during the 5-cycle repeated batch process

**FIGURE 5:**
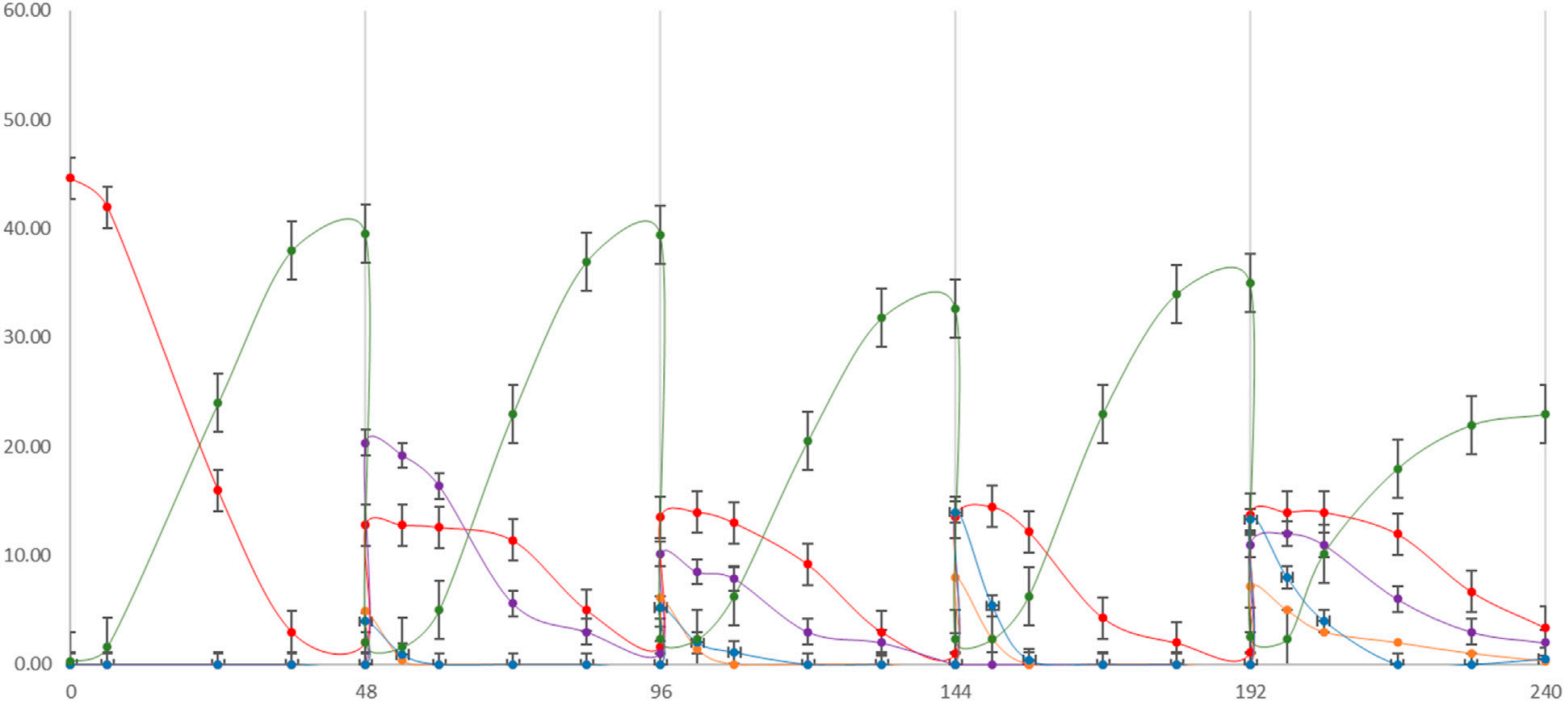
cycle repeated batch fermentation process: first cycle (0–48 h) SCW + MSL (10%); second cycle (48–96 h) SCW 30% + peach pomace 70%; third cycle (96–144 h) SCW 30% + melon pomace; fourth cycle (144–192 h) SCW 30% + 70% pear pomace; fifth cycle (192–240 h) SCW 30% + 70% apple pomace. LA production (green line) along with lactose (red line), sucrose (purple line), fructose (blue line), and glucose (orange line) depletions were monitored.

Repeated batch fermentation was used as the strategy based on the findings from the previously tested single substrate fermentation. The aim here was to simulate an all-season process using different FWs in series based on their specific seasonal availabilities. SCW is considered to be annually available and was consequently added in all cycles at 30% along with MSL at 10% as the nitrogen source. The overall process duration was 240 h, comprising 48 h for each cycle until complete depletion of the corresponding carbon source (Figure 5).

An inoculum of 10% fermentation broth (FB) was maintained for each batch from the previous cycle, based on the repeated batch strategy. At the beginning of fermentation, a lag phase of approximately 6 h was observed, which reduced to 1–2 h in the following batch. LA yield, substrate uptake, and LA volumetric (Q_LA_) productivity in each batch cycle were recorded (Table 5). A total quantity of 180.56 g/L of LA was produced. In total, approximately 830 g of LA was cumulatively recovered from 4.6 L of the FB. The overall LA yields from the total sugar (TS) and consumed sugar (CS) were 88.0% and 92.9%, respectively; the average LA volumetric productivity expressed as Q_LA_ was estimated to be 0.88 g/L∙h. Although lactose, sucrose, glucose, and fructose are all present as substrates, the order of sugar utilization by *L. casei* is glucose ≥ fructose > sucrose > lactose, as has been generally reported for other Lactobacilli in complex media (Srinivas et al., 1990). The fifth batch cycle with the apple pomace substrate was confirmed to have the worst fermentation performance, similar to that in the single substrate process; its yield and productivity were the lowest, in addition to the low sugar consumption and LA production.

**TABLE 5:** Repeated batch fermentation parameters for each cycle. TS, total sugars; S_uptake_, substrate uptake percentage; LA concentration, LA yields on TS (Y_LA/TS_) and consumed sugar (CS) (Y_LA/CS_); and volumetric productivity (Q_LA_) were calculated.

#Batch	TS (g/L)	S_uptake_ (%)	LA (g/L)	Y_LA/TS_ (%)	Y_LA/CS_ (%)	Q_LA_ (g/L⋅h)
1	44.66	95.5	39.50	88.4	92.6	0.82
2	42.61	93.9	39.63	86.8	98.7	1.12
3	35.53	97.2	33.75	89.5	97.6	0.94
4	34.24	96.8	33.09	89.8	98.6	0.92
5	35.01	84.8	23.65	64.4	81.8	0.64

### 3.4 Fermentative performance of pretreated apple pomace

**FIGURE 6:**
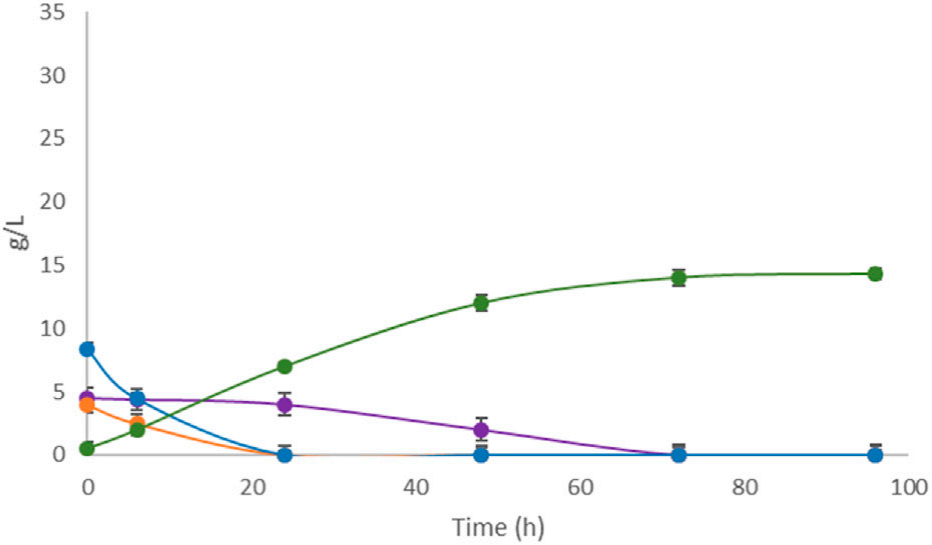
Single-substrate LA fermentation by *L. casei* with filtered apple pomace as the carbon source. Concentrations of LA (green line) along with sucrose (purple line), glucose (orange line), and fructose (blue line) were monitored for 96 h.

**FIGURE 7:**
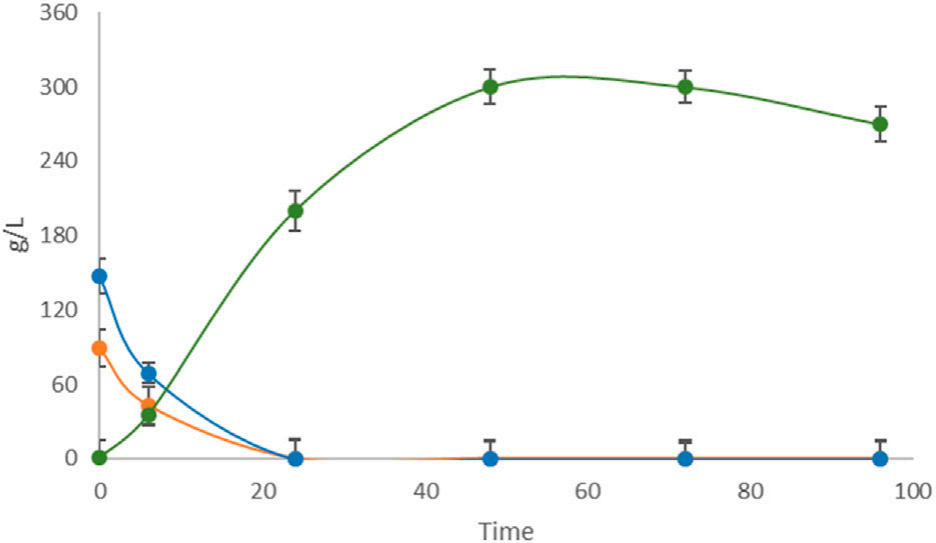
Single-substrate LA fermentation by *L. casei* with hydrolyzed apple pomace as the carbon source. Concentrations of LA (green line) along with sucrose (purple line), glucose (orange line), and fructose (blue line) were monitored for 96 h.

Untreated apple pomace showed low fermentative performance in the Erlenmeyer flask-scale fermentation as well as in the controlled fermenter, unlike the other FWs in this study. Therefore, two different pretreatments, i.e., mechanical filtration and enzymatic hydrolysis, were attempted. The pretreated samples were fermented by *L. casei* under the conditions reported in Section 3.1 as mechanically filtered apple pomace (Figure 6) and enzymatically hydrolyzed apple pomace (Figure 7).

Complete sugar depletion occurred in the filtered medium, with a LA yield of 85.00%, which was significantly improved fermentation performance over the untreated sample. Otherwise, fermentation of the hydrolyzed apple pomace sample resulted in a yield of 115% on the initial amount of glucose and fructose, with a production of 270 g/L of LA on 235 g/L of TS, indicating that hydrolysis produced an unquantified amount of fermentable monosaccharides other than glucose and fructose, such as galactose and mannose derived from the hydrolysis of glucans, which were then used by *L. casei* as substrates for LA production. Finally, a second round of the 5-cycle repeated batch strategy was implemented, including the hydrolyzed apple pomace as the carbon source in the final step (data not shown). The fifth step was allowed to last 96 h, which resulted in volumetric productivities of 2.81 g/L∙h and 1.32 g/L∙h for the fifth cycle and entire process, respectively. The total amount of LA produced in the 5-cycle repeated batch process using different FWs in sequence was 397.1 g/L over 288 h.

## 4 Discussion

The main objective of this work was to study the use of FWs and SCW as substrates to establish an alternative and a more sustainable method of obtaining LA through the fermentative approach while reducing the environmental impacts of these residues. To assess whether the FWs and SCW could be efficiently fermented, the fermentative performances of the *L. casei* strain were first evaluated in media containing SCW, peach, melon, pear, and apple pomace separately. *L. casei* exhibits homofermentative metabolism of glucose, which primarily produces LA. Under anerobic conditions, one molecule of glucose is catabolized to two molecules of pyruvate via the EMP pathway. These pyruvate molecules are subsequently reduced to LA by lactate dehydrogenase (LDH) utilizing NADH^+^ H^+^ that generates two molecules of ATP per glucose molecule. Fructose metabolism in *L. casei* follows a similar route; after fructose is converted to fructose 1,6-bisphosphate, it is integrated into the glycolytic pathway, ultimately undergoing the same biochemical steps as glucose metabolism (Cui and Qu, 2021). Recent analyses of sugar utilization have demonstrated that *L. casei* can ferment a broad spectrum of monosaccharides and disaccharides, including glucose, fructose, galactose, mannose, ribose, sorbose, lactose, sucrose, maltose, trehalose, and cellobiose (Viana et al., 2000).

LA is one of the most important chemicals and a keystone product in industrial biotechnology; its market demand is increasing progressively and is not predicted to decline in the near future. However, the high costs of the raw materials and fermentation–separation processes have severely limited LA production (Ojo and de Smidt, 2023). For a low-priced commodity like LA, production from pure sugars is undoubtedly unsustainable for both environmental and economic reasons. However, two strategies are suggested to reduce the bioprocesses costs, i.e., selecting cheaper raw materials for fermentation and upgrading the production technologies. Several studies have focused on the development of consolidated bioprocesses for direct fermentation of complex and recalcitrant biomass, such as lignocellulose, through the design of specific microbial consortia and exploration of recombinant cellulolytic strains by metabolic engineering (Singhania et al., 2022). In both cases, the approaches for LA production remain highly challenging and far from being applicable at the industrial scale (Huang et al., 2023).

The results obtained from the single substrate fermentations suggest the feasibility of using *L. casei* to ferment FWs and SCW in the absence of inhibitor compounds that can have negative effects on fermentation. Moreover, pretreatments other than mixing and rough filtration appear to be necessary before the fermentations. It was confirmed at all FWs except for apple pomace, where the particular gel-like consistency of the slurry and presence of fibrous material necessitated additional mechanical filtration and hydrolysis, could be used to obtain significant LA yields. Theoretically, enzymatic hydrolysis treatments could be applied to all FWs with the prospect of increasing the fermentable sugars and consequently the LA from the given masses. However, in this study, pretreatments were relegated to a minimum and used only where strictly necessary to create a low-cost fermentation system. Pretreatments are one of the major cost categories in agri-food waste biorefinery processes, and the costs often involved in pretreatments are equivalent to the cost savings obtained by using waste biomass (Macedo et al., 2020). In single substrate fermentation, the rapid development of an acidic environment due to the lack of pH control is preserved from contamination, thus avoiding sterilization. On the other hand, the inhibitory effects of the end-product due to LA accumulation in the fermentation system can reduce the yield and productivity while increasing the fermentation time. Such inhibitory effects on the metabolic activity can be overcome by scaling-up to the fermenter, using a pH control system, and periodically removing the end-product accumulated in the medium. In the repeated batch experiments, controlling the pH can have significant effects on both LA production and sugar depletion. The repeated batch fermentation strategy was chosen here because of its advantages in terms of low investment costs, simple control and operations, and easy-to-maintain complete sterilization than the continuous strategy (Reddy et al., 2016). The repeated batch experiments conducted with various renewable substrates demonstrate the feasibility of producing large amounts of LA from different raw materials. Moreover, using seasonally available FWs as the substrates with repeated batches permits separate feeding of the fermenter with the various substrates based on their availabilities.

Many studies have highlighted the advantages of continuous and batch-fed fermentation modes on LA productivity, which are attributable to lower product inhibition, since LA is constantly removed from the bioreactor (Ahring et al., 2016; Olszewska-Widdrat et al., 2020). In this case, we applied simplicity of operation and process management as the criteria, especially without the obligation to maintain sterility. In fact, our experiments provide a small-scale simulation of the operations in a local biorefinery, which is located as close as possible to the waste production site and is supplied with wastes from primary agri-food manufacturing operations. Starting from the beginning of the seasonal campaign, with the pomace of early summer fruits to late summer and early fall productions, LA can be produced uninterruptedly for several months based on the substrate availabilities. In our simulated production process, each batch cycle was supplied with approximately 35–40 g/L of sugars, which were almost totally depleted in 48 h with 94% average yield and 0.9 g/L∙h productivity. With the hydrolyzed apple pomace, the average yield and productivity increased to about 99% based on the sugar consumed and 1.32 g/L∙h, respectively. This yield took into account the overall fermentable sugars, including the quantity released by hydrolysis. The total amount of LA produced in the five cycles was 397.1 g/L over 288 h and without apparent contamination.

Biomass concentration was not reported here, and LA production was instead used as an indicator of the good fermentation course. It is well known that LA production is partially associated with growth. After the lag phase, the metabolite production rate becomes proportional to the active biomass concentration in the reactor (Alvarez et al., 2010). Some of the sugars available in the FWs are glucose, fructose, sucrose, and lactose. Glucose and fructose are the most favorable types of sugars available for assimilation by *L. casei* for producing LA. Similar to the other LAB, *L. casei* follows a hierarchical pattern of sugar utilization. In particular, glucose is consumed faster than fructose because it has been demonstrated that the enzymes for glucose metabolism are constitutive, whereas fructose metabolism requires induction of certain enzymes in the initial stages before the sugars enter the metabolic pathway. Analogous reasons can also be used to explain how *L. casei* has a slightly higher preference for sucrose than lactose. Kachhy et al. (1977) and Mital et al. (1973) found that galactosidases and fructofuranosidases, which are required for lactose and sucrose hydrolysis, are inducible and constitutive enzymes, respectively. Moreover, the complexity of galactose metabolism, as one of the monomers of lactose, is reported to play a role in the slower utilization of lactose in *L. casei* (Srinivas et al., 1990). It is worth noting that some authors have reported that addition of sucrose to milk fermentation stimulates LA production by the lactobacilli (Nurhartadi et al., 2017) as well as the addition of glucose and fructose (Zielińska et al., 2021), confirming our results.

In this study, to further reduce the manufacturing costs of LA, MSL supplementation is proposed to replace expensive components like yeast extract or peptones. Li et al. (2006) evidenced that MSL is a good choice even if it does not fully substitute the vitamin and salt contents; they reported that the final LA concentrations in MSL-supplemented media were almost equal to those achieved with yeast extract, with only a slight decrease in productivity. The use of SCW as a fermentation substrate has been recently recommended by both the European Commission and FAO (Papirio et al., 2020). To the best of our knowledge, the results presented here are comparable to those reported by other authors (Table 6).

**TABLE 6:** Non-exhaustive comparison of the LA fermentation performances by *L. casei* on the raw substrates. S_uptake_, substrate uptake percentage; LA yields on TS (Y_LA/TS_) and consumed sugar (CS) (Y_LA/CS_); and volumetric productivity (Q_LA_) are reported, when available.

Principal carbon source	S_uptake_ (%)	Y_LA/TS_ (%)	Y_LA/CS_ (%)	Q_LA_ (g/L⋅h)	Reference
Deproteinized whey	90	55.5		2.5	Roukas and Kotzekidou (1996)
Ovine SCW	100	—	86.5	0.71	Secchi et al. (2012)
Whey permeate	98	90	—	—	Fitzpatrick et al. (2001)
MSL-supplemented soybean meal	—	89.7	—	1.69	Li et al. (2006)
Mixed food waste	—	92	—	2.5	Kwan et al. (2016)

Although our findings confirm that FWs and SCW have great potential as raw materials in biorefineries for producing value-added materials, their large-scale uses still show potential risks of economic and environmental unfeasibility, which are associated with their logistics, supply, and high perishability (Jiménez-Moreno et al., 2020). In fact, one of the major bottlenecks limiting the wide use of agri-food wastes at present is transportation from the site of production to the biorefinery, which accounts for a considerable proportion of the environmental impact (Ögmundarson et al., 2020). The option of building small local biorefineries, however, represents the problem of overall economic profitability that usually privileges large-scale plants.

## 5 Conclusion

Microbial fermentation of LA using agricultural and food wastes has emerged as a promising alternative to traditional chemical synthesis. This approach not only adds value to waste materials but also aligns with sustainable and ecofriendly production practices. In this study, fermentation of SCW, an annually available biomass, along with seasonally available FWs such as peach, melon, pear, and apple pomace, which are byproducts of the food processing industry, by *L. casei* was explored. Feedstock pretreatments were reduced and sterilization was avoided whenever possible to ensure that the process remained low-cost. Only apple pomace required preliminary enzymatic hydrolysis before fermentation. A repeated batch process was set up, yielding a maximum of 397.1 g/L of LA over 288 h, resulting in a volumetric productivity of 1.32 g/L∙h. However, for this process to be viable at a higher scale, further studies focused on upstream, scaling up, and downstream operations as well as economic feasibility analyses are essential. These findings represent a significant step forward in the sustainable production of LA, offering a practical solution for waste management while creating valuable bioproducts.

## Data Availability

The raw data supporting the conclusions of this article will be made available by the authors without undue reservation.
